# Information Entropy of Tight-Binding Random Networks with Losses and Gain: Scaling and Universality

**DOI:** 10.3390/e21010086

**Published:** 2019-01-18

**Authors:** C. T. Martínez-Martínez, J. A. Méndez-Bermúdez

**Affiliations:** Instituto de Física, Benemérita Universidad Autónoma de Puebla, Apartado Postal J-48, Puebla 72570, Mexico

**Keywords:** information entropy, Erdős–Rényi graphs, random matrix theory, scaling laws

## Abstract

We study the localization properties of the eigenvectors, characterized by their information entropy, of tight-binding random networks with balanced losses and gain. The random network model, which is based on Erdős–Rényi (ER) graphs, is defined by three parameters: the network size *N*, the network connectivity α, and the losses-and-gain strength γ. Here, *N* and α are the standard parameters of ER graphs, while we introduce losses and gain by including complex self-loops on all vertices with the imaginary amplitude iγ with random balanced signs, thus breaking the Hermiticity of the corresponding adjacency matrices and inducing complex spectra. By the use of extensive numerical simulations, we define a scaling parameter ξ≡ξ(N,α,γ) that fixes the localization properties of the eigenvectors of our random network model; such that, when ξ<0.1 (10<ξ), the eigenvectors are localized (extended), while the localization-to-delocalization transition occurs for 0.1<ξ<10. Moreover, to extend the applicability of our findings, we demonstrate that for fixed ξ, the spectral properties (characterized by the position of the eigenvalues on the complex plane) of our network model are also universal; i.e., they do not depend on the specific values of the network parameters.

## 1. Introduction

Independently of the field, classification, or application, a commonly-accepted mathematical representation of a network or graph is the adjacency matrix. The adjacency matrix A of a simple non-directed network (a simple network is a network not having multiple edges or self-edges) is the matrix with elements $A_{ij}$ defined as [1]:

(1) $$A_{ij}=\left\{\begin{array}{cc} 1\; & \mathrm{if}\;\mathrm{there}\;\mathrm{is}\;\mathrm{an}\;\mathrm{edge}\;\mathrm{between}\;\mathrm{vertices}\;i\;\mathrm{and}\;j,\\ 0\; & \mathrm{otherwise}. \end{array}\right.$$

This prescription produces N×N symmetric sparse matrices with zero diagonal elements, where *N* is the number of vertices of the corresponding network. The sparsity of A is quantified by the parameter α, which is the fraction of non-vanishing off-diagonal adjacency matrix elements. Vertices are isolated when α=0, whereas the network is fully connected for α=1. Once the adjacency matrix of a network is constructed, it is quite natural to ask about the properties of its eigenvalues and eigenvectors, which is the main subject of this paper. As commonly used, we refer to the properties of the eigenvalues and eigenvectors of the adjacency matrix as the properties of the eigenvalues and eigenvectors of the respective network.

### 1.1. Network Model with Losses and Gain

First, we want to recall that there is a one-to-one correspondence between the adjacency matrix A of Equation (1) and the Hamiltonian matrix H of a ξ-dimensional solid, described by Anderson’s tight-binding model [2] with zero on-site potentials ($H_{ii}=0$) and constant hopping integrals ($H_{ij}=1$). Here, ξ=αN is proportional to the average non-zero off-diagonal adjacency matrix elements per matrix row and, therefore, may be regarded as the effective dimension of a tight-binding random network represented by A, as discussed in [3] from a Random Matrix Theory (RMT) point of view. This correspondence enables the direct application of studies originally motivated by physical systems, represented by sparse random Hamiltonian matrices, to complex networks. Tight-binding models are widely used in solid state physics to study the electronic properties of systems composed by atoms (molecules, potentials, or sites; in more general terms) whose electrons are tightly bound to the atoms they belong to, so they have limited interaction with neighbor atoms; see, e.g., [4]. Moreover, recently, tight-binding models have also been studied on random and regular graphs; see some examples in [5,6,7,8,9,10,11,12,13].

The tight-binding random network model we shall use in our study is defined as follows. Starting with the *standard* Erdős–Rényi (ER) network, we add to it self-edges and further consider all edges to have random strengths. Our main motivation to include weights, particularly random weights, in the standard ER model is to retrieve well-known random matrices in the appropriate limits in order to use RMT results as a reference (see below). Moreover, we would like to note that in realistic graphs, vertices and edges may not be equivalent (i.e., the graph might be composed of different agents, where different pairs interact with different intensities); therefore, their corresponding adjacency matrices are not just binary. In this sense, random weights can be considered as a limit case where all vertices and edges in a graph are different. Indeed, random weights have been used in other complex network models; see some examples in [14,15]. We have named this model as the ER *fully*-random network model [5,16,17]. The sparsity α is defined as the fraction of the N(N−1)/2 independent non-vanishing off-diagonal adjacency matrix elements. Then, as in the *standard* ER model, the ER *fully*-random network model (whose adjacency matrices come from the ensemble of N×N sparse real symmetric matrices) is characterized by the parameters *N* and α. In this study, we add the imaginary amplitude ±iγ to the self-edge weights of the ER *fully*-random network model such that the corresponding adjacency matrices are defined as:

(2) $$A_{ij}=\left\{\begin{array}{cc} \epsilon_{ij}+{\left(-1\right)}^{i}i\gamma \; & \mathrm{for}\;i=j,\\ \epsilon_{ij}/\sqrt{2}\; & \mathrm{if}\;\mathrm{there}\;\mathrm{is}\;\mathrm{an}\;\mathrm{edge}\;\mathrm{between}\;\mathrm{vertices}\;i\;\mathrm{and}\;j,\\ 0\; & \mathrm{otherwise}. \end{array}\right.$$

Here, $\epsilon_{ij}=\epsilon_{ji}$ are statistically-independent random variables drawn from a normal distribution with zero mean and variance one. Note that the term ±iγ, with γ≠0, makes the adjacency matrix of the tight-binding random network model non-Hermitian, which, in turn, has complex eigenvalues and eigenvectors. According to this definition, a diagonal random matrix is obtained for α=0 and γ = 0 (Poisson case), whereas the Gaussian Orthogonal Ensemble (GOE) is recovered when α=1 and γ=0. The GOE is a random matrix ensemble formed by real symmetric random matrices A whose entries are statistically-independent random variables drawn from a normal distribution with zero mean and variance $\langle |A_{ij}{|}^{2}\rangle =\left(1+\delta_{ij}\right)/2$; see, e.g., [18]. The GOE is commonly used to statistically represent Hamiltonian matrices corresponding to complex, chaotic, or disordered systems having time-reversal invariance.

The random network model with the adjacency matrix of Equation (2) is inspired by non-Hermitian Hamiltonians describing open or scattering systems, systems interacting with an environment, or active materials. Within the effective non-Hermitian Hamiltonian approach, such opening or interaction is modeled by adding complex terms to the main diagonal of the Hamiltonian of the system of interest [19,20,21,22,23]. Indeed, in tight-binding systems, the on-site term ±iγ represents losses (iγ) and gain (−iγ). Moreover, the term ±iγ allows adding losses and gain to tight-binding systems locally by adding this term to selected sites (in regular arrays, the addition of the term iγ to border sites is commonly used to study scattering and transport properties; see, e.g., [24,25,26]), globally by adding this term to all sites in the system (in linear chains, the addition of the term iγ to all sites has been used to represent a system coupled to a common decay channel; see, e.g., [27,28]), and in a balanced way by adding iγ to all sites with the same proportion of plus and minus signs (the addition of alternating iγ and −iγ terms to the sites of one-dimensional non-disordered arrays produces PT-symmetric wires; see, e.g., [29,30]). In our model, we choose the latter setup, where balanced implies that the network is formed by an even number of vertices. Our main motivation to choose a balanced loss-and-gain setup is to limit the number of parameters of the model, since a non-balanced setup would require including the loss-to-gain ratio as a parameter. Furthermore, since the vertices of our network are not ordered, the balanced loss and gain is effectively introduced randomly to the network. This is in contrast to PT-symmetric systems [29], where loss and gain alternate periodically. Thus, in our model, γ is the loss-and-gain strength.

Therefore, our random network model corresponds to tight-binding random networks with random on-site potentials, random hopping integrals, and random on-site loss and gain. Our random network model depends on three parameters: the network size *N*, the network connectivity α, and the losses-and-gain strength γ.

### 1.2. Previous Work

As precedents, we can mention that we have already studied some spectral [16,17], eigenvector [16], and transport [5] properties of ER-type random networks with a special focus on universality, from a random matrix theory (RMT) point of view. Moreover, we have also performed scaling studies on other random networks models, such as multilayer and multiplex networks [15,31] and random-geometric and random-rectangular graphs [32]. In particular, for ER fully-random networks, we have shown that [16] the average information entropy $\langle S\rangle$ (to be defined below) is a function of the average degree ξ=αN. Moreover, $\langle S\rangle$ describes the delocalization transition of the network model well: (i) for $\xi \overset{<}{∼}2$, where $\langle S\rangle \approx 0$, the eigenvectors are practically localized; hence, the delocalization transition takes place around ξ≈2 (for which $\langle S\rangle$ becomes larger than zero, meaning that the corresponding eigenvectors have more than only one principal component), which is close to previous theoretical and numerical estimations [33,34,35,36]; and (ii) for ξ>200, where $\langle S\rangle \approx {\langle S\rangle }_{\mathrm{GOE}}\equiv \;S_{\mathrm{GOE}}\approx \ln\left(N/2.07\right)$, the eigenvectors are practically random and fully extended. Here, $S_{\mathrm{GOE}}$ is the entropy of the eigenvectors of the GOE, i.e., random eigenvectors with Gaussian-distributed amplitudes. Thus, the study of [16] provides a tool to predict the localization properties of the eigenvectors of ER-type random networks once the parameter ξ is known.

Thus, in Section 2, we study some eigenvector and eigenvalue properties of the ER tight-binding random networks with balanced losses and gain, corresponding to the non-Hermitian adjacency matrices of Equation (2), focusing on scaling and universality from an RMT point of view.

It is fair to say that there are several works in the literature that apply RMT approaches to the study of spectral and eigenvector properties of non-Hermitian sparse matrices, in some cases already applied to graphs or network models; see for example [37,38,39,40,41,42,43,44,45,46].

## 2. Results

### 2.1. Scaling of Information Entropy

In order to characterize quantitatively the complexity, and in specific cases the fractality, of the normalized eigenvectors Ψ of random matrices (and of Hamiltonians corresponding to disordered and quantized chaotic systems), the Rényi entropies are widely used:

(3) $$R_{q}=\frac{1}{1-q}\ln\left(\sum_{n=1}^{N}{\left(\rho_{n}\right)}^{q}\right)\;.$$

Here, the subindex *n* refers to the $n^{\mathrm{th}}$ eigenvector component, and $\rho_{n}\equiv {\left|\Psi_{n}\right|}^{2}$ form the discrete probability distribution $P=(\rho_{1},\ldots ,\rho_{N})$ associated with the eigenvector Ψ (where |·| stands for the modulus of a complex number); with $\rho_{n}\geq 0$ and $\sum_{n=1}^{N}\rho_{n}=1$. In our study, we use the information entropy (given by Equation (3) in the limit q→1):

(4) $$S=-\sum_{n=1}^{N}\rho_{n}\ln\rho_{n}.$$

Note that the minimal value of *S*, S=0, is obtained when only one component in the eigenvector Ψ concentrates all the probability; while the maximal value of *S*, S=lnN, is approached when the probability is evenly distributed over the eigenvector: $\rho_{n}=1/N$ for all *n*. Any other possible configuration of probabilities $\rho_{n}$, including the eigenvectors of the GOE, provides 0<S<lnN. Therefore, the exponential of *S* is known to be a good measure of eigenvector localization [47], since it provides the number of principal components of an eigenvector in a given basis. That is, when *S* = 0, the eigenvector has only one principal component, exp(S)=1, so it is localized; while it is fully extended, exp(S)=N, when S=lnN. Here, we refer to the principal components of an eigenvector as the eigenvector components having the largest amplitudes. In fact, *S* has been already used to characterize the eigenvectors of adjacency matrices of several random network models (see some examples in [15,16,48,49,50,51]).

With Definition (4), when α=0 for any γ≥0, since the eigenvectors of the (diagonal) adjacency matrices of our random network model have only one non-vanishing component with the magnitude equal to one, then $\langle S\rangle =0$. On the other hand, for α=1 and γ=0, the GOE is reproduced, and $\langle S\rangle =S_{\mathrm{GOE}}$; i.e., the random eigenvectors extend over the *N* available vertices in the network. We note that for α=1 and γ≠0, our random network model does not reproduce the GOE and $\langle S\rangle \neq S_{\mathrm{GOE}}$; however, we observe that $\langle S\rangle \approx S_{\mathrm{GOE}}$, so we use $S_{\mathrm{GOE}}$ as the reference information entropy.

Below, we use exact numerical diagonalization to obtain the eigenvectors $\Psi^{m}$ and eigenvalues $\lambda_{m}$ (m=1…N) of the adjacency matrices of large ensembles of tight-binding random networks characterized by *N*, α, and γ. Then, we average over all eigenvectors of an ensemble of adjacency matrices of size *N* to compute $\langle S\rangle$. We have verified that our conclusions are not modified when we restrict the averages to a fraction of the eigenvectors around the band center, which is a prescription commonly used in RMT studies.

**Figure 1:**
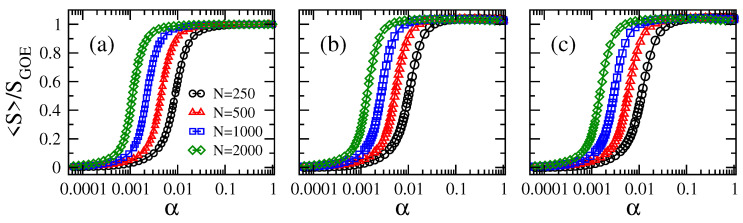
Average information entropy $\langle S\rangle$ normalized to $S_{\mathrm{GOE}}\approx \ln\left(N/2.07\right)$ as a function of the connectivity α of Erdős–Rényi tight-binding random networks (of sizes ranging from N=250–2000) with balanced losses and gain with strength γ. (**a**) γ=0.01, (**b**) γ=1, and (**c**) γ=2. Each symbol was computed by averaging over $10^{6}$ eigenvectors.

**Figure 2:**
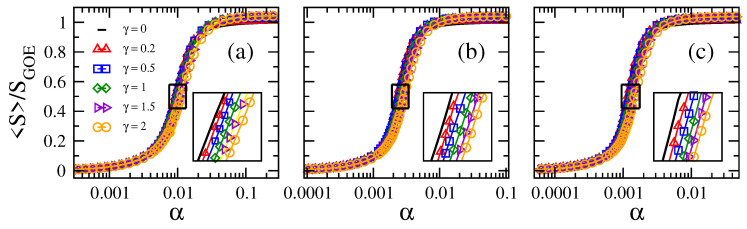
Average information entropy $\langle S\rangle$ normalized to $S_{\mathrm{GOE}}\approx \ln\left(N/2.07\right)$ as a function of the connectivity α of Erdős–Rényi tight-binding random networks of size *N* with different loss-and-gain strengths γ. (**a**) N=250, (**b**) N=1000, and (**c**) N=4000. Insets: enlargements of the boxes around the localization-to-delocalization transition point in main panels. Each symbol was computed by averaging over $10^{6}$ eigenvectors.

In Figure 1 and Figure 2, we show the average information entropy $\langle S\rangle$, normalized to $S_{\mathrm{GOE}}$, as a function of the connectivity α for the adjacency matrices of ER tight-binding random networks with balanced losses and gain. We observe that the curves of $\langle S\rangle /S_{\mathrm{GOE}}$, for any combination of *N* and γ, have a very similar functional form as a function of α: the curves $\langle S\rangle /S_{\mathrm{GOE}}$ show a smooth transition from approximately zero (localized regime) to approximately one (delocalized regime) when α increases from α∼0 (mostly isolated vertices) to one (fully-connected graphs).

From Figure 1, for fixed γ, we observe that the larger the network size *N*, the smaller the value of α needed to approach the delocalized regime. Furthermore, note that the curves of $\langle S\rangle /S_{\mathrm{GOE}}$ vs. α are shifted to the left on the α-axis for increasing *N*. All this panorama is in accordance with the case γ=0, as shown in [16]. In contrast, for fixed *N*, the curves of $\langle S\rangle /S_{\mathrm{GOE}}$ vs. α are displaced to the right on the α-axis for increasing γ; clearly seen in the insets of Figure 2. As a reference, we include the case γ=0 as black full lines in all panels of Figure 2. Moreover, the fact that these curves, plotted in semi-log scale, are just shifted on the α-axis when tuning *N* or γ makes us forecast the existence of a scaling parameter that depends on both *N* and γ. In order to look for the scaling parameter, we first define a quantity to characterize the position of the curves $\langle S\rangle /S_{\mathrm{GOE}}$ on the α-axis: indeed, we choose the value of α, which we label as $\alpha^{*}$, for which $\langle S\rangle /S_{\mathrm{GOE}}\approx 0.5$. Notice that $\alpha^{*}$ characterizes the localization-to-delocalization transition of the eigenvectors of our network model.

**Figure 3:**
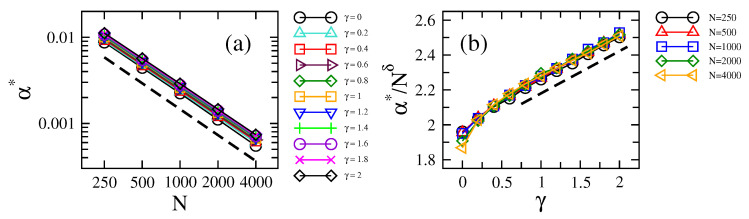
Localization-to-delocalization transition point $\alpha^{*}$ (defined as the value of α for which $\langle \;S\;\rangle /S_{\mathrm{GOE}}\;\approx \;0.5$) as a function of (**a**) the network size *N* (for several values of γ) and (**b**) the loss-and-gain strength γ (for several values of *N*). In (**b**), we set δ to −0.98. Dashed lines in (**a**) and (**b**) proportional to $N^{-0.98}$ and γ, respectively, are plotted to guide the eye; see Equations (5) and (6).

Then, in Figure 3a,b, we present the localization-to-delocalization transition point $\alpha^{*}$ as a function of *N* and γ, respectively. On the one hand, the linear trend of the data (in log-log scale) in Figure 3a implies a power-law relation of the form:

(5) $$\alpha^{*}=CN^{\delta }\;.$$

In fact, Equation (5) provides very good fittings to the data (the values of the fitting parameters are reported in Table 1). Note that δ≈−0.98 for all γ>0, a slight difference with the case γ=0 where δ≈−1; see also [16]. On the other hand, in Figure 3b, we plot the ratio $\alpha^{*}/N^{\delta }$, with δ=−0.98, as a function of γ. With this, we already take into account the scaling stated in Equation (5), which, at the same time, allows us to examine the dependence of $\alpha^{*}$ on γ more easily. Indeed, for γ>0.4, we conclude that:

(6) $$\frac{\alpha^{*}}{N^{\delta }}=C\approx 2+\frac{\gamma }{4}.$$

**Figure 4:**
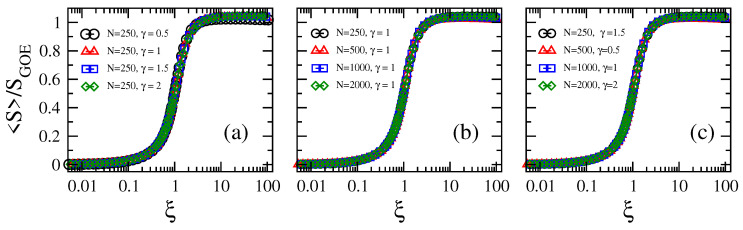
Average Shannon entropy $\langle S\rangle$ normalized to $S_{\mathrm{GOE}}$ as a function of the scaling parameter ξ (see Equation (7)) of Erdős–Rényi tight-binding random networks with losses and gain. (**a**) N=250 for different values of loss-and-gain strength γ, (**b**) γ=1 for different network sizes *N*, and (**c**) different combinations of *N* and γ.

Therefore, by plotting again the curves of $\langle S\rangle /S_{\mathrm{GOE}}$ now as a function of the connectivity divided by the localization-to-delocalization transition point,

(7) $$\xi \equiv \frac{\alpha }{\alpha^{*}}\;,$$

we observe that curves for different parameter combinations (N,γ) collapse on top of a universal curve; i.e., a curve that depends on the parameter ξ only; see Figure 4. This means that once the ratio ξ is fixed, no matter the graph size and the loss-and-gain strength, the information entropy of the eigenvectors is also fixed.

### 2.2. Eigenvalue Properties

Once we have found that ξ (see Equations (6) and (7)) is the parameter that scales the eigenvector properties (characterized by their information entropy) of our model of random networks with losses and gain, we believe that other properties (i.e., spectral properties) of the network model may also be scaled by the same parameter. Thus, in the following, we validate our surmise by analyzing the corresponding eigenvalues.

Recall that for γ=0, the adjacency matrices of our random network model are Hermitian and the corresponding spectra are real. For any γ>0, the adjacency matrices become non-Hermitian and their eigenvalues λ are complex numbers.

**Figure 5:**
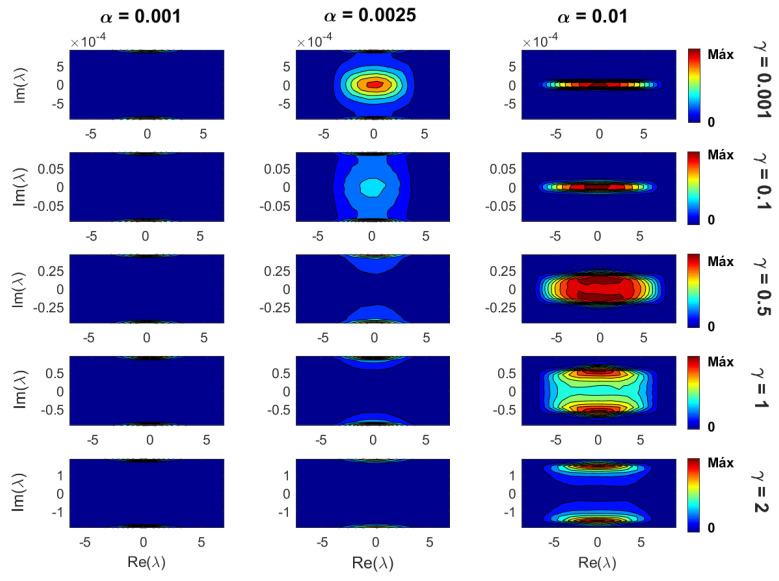
Density plots of eigenvalues λ in the complex plane for several combinations of sparsity α and loss-and-gain strengths γ. The network size was set to N=1000. The sparsity increases from left to right, while the loss-and-gain strength from top to bottom. To construct each density plot, $10^{6}$ eigenvalues were used.

Now, in Figure 5, we show density plots (in the complex plane) of the eigenvalues λ of Erdős–Rényi tight-binding random networks with losses and gain for several parameter combinations. In this figure, we can clearly see the competition of the two main parameters of the model: the sparsity α and the loss-and-gain strength γ (for fixed *N*). On the one side, for small α (i.e., mostly isolated vertices), the main diagonal of the adjacency matrices dominates and the imaginary part of the corresponding eigenvalues is approximately equal to ±iγ; see Figure 5 (left panels). That is, the eigenvalues form two thin clouds around ±iγ. On the other side, for large α (i.e., highly-connected graphs), the density of off-diagonal elements of the adjacency matrices is also large, and the corresponding eigenvalues form a cloud with center at the origin of the complex plane that gets wider for increasing γ; see Figure 5 (right panels) with 0.001<γ<0.5. Moreover, for γ≈1, this cloud splits into two clouds that separate further from the real axis for even larger values of γ; see Figure 5 (right panels) with γ>0.5. It is remarkable that the cloud splits for γ≈1, since it corresponds to the super-radiance transition value reported for full random matrices [52], one-dimensional disordered tight-binding wires [26,27,53,54,55], and random many-body systems [56].

The super-radiance transition is a phase transition that occurs, as a function of the coupling strength, in quantum systems coupled to common decay channels; see, e.g., [20,21,22,23,57]. It was originally predicted by the Dicke model of super-radiance [58]. In very general terms, this transition occurs at a given coupling strength above which a number of internal states (eigenvalues) acquire decay widths (imaginary part of the eigenvalues) proportional to the coupling strength. Thus, even though a more detailed analysis is necessary, we can assume that the splitting of the density plots of eigenvalues in the complex plane at γ≈1 (as observed in Figure 5) is a signature of the super-radiance transition in our tight-binding random network model.

Finally, for moderate values of α, as reported in Figure 5 (central panels), the combination of the two situations described above occurs: for small γ, the eigenvalues form three clouds in the complex plane, two thin ones close to ±iγ, and a third one with the center at the origin of the complex plane; for increasing γ, the middle cloud gets wider and splits into two clouds that, for large enough γ, merge with the thin clouds at ±iγ.

**Figure 6:**
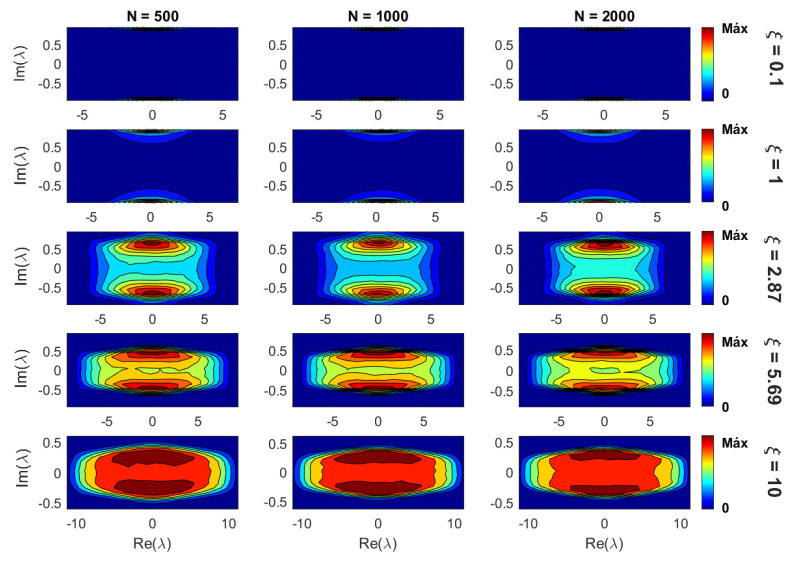
Density plots of eigenvalues λ in the complex plane for three network sizes *N* (500, 1000, and 2000) and increasing values of ξ (from top to bottom). To construct each density plot, $10^{6}$ eigenvalues were used.

Notice that the panorama shown in Figure 5 (where networks of size N=1000 were used), even though it is valid for any *N*, will be shifted for different network sizes; as can be inferred from the information entropy of the eigenvectors reported in Figure 1. Moreover, the scaling analysis made in the previous subsection allowed us to define the scaling parameter ξ that fixes the eigenvector properties of our random network model, as shown in Figure 4. Therefore, in Figure 6 we present density plots of eigenvalues for three network sizes and increasing values of ξ (from top to bottom). It is clear from Figure 6 that once ξ is fixed, the density of eigenvalues in the complex plane is (statistically) the same for different parameter combinations. Thus, we validate that the eigenvalue properties of our model are also scaled with the parameter ξ.

## 3. Summary

In this paper, we have numerically studied the eigenvector and eigenvalue properties of the adjacency matrices of tight-binding random networks with balanced losses and gain. In particular, we focused on scaling and universality from a random matrix theory point of view. We would like to stress that even though we already have some previous experience with scaling studies of random network models (see, e.g., [5,15,16,17,32]), this is the first time we apply this technique to non-Hermitian adjacency matrices.

Specifically, we have considered Erdős–Rényi tight-binding random networks with self-loops (where all non-vanishing adjacency matrix elements are Gaussian random variables) and add the imaginary term ±iγ to the weights of all vertices to emulate losses (iγ) and gain (−iγ). We assume balanced losses and gain, so that we include the same number of positive and negative terms iγ. This implies the number of vertices in the network to be an even number. Thus, our random network model depends on three parameters: the network size *N*, the network connectivity α, and the losses-and-gain strength γ.

First, by the proper scaling analysis of the information entropy of the eigenvectors of the adjacency matrices of our random network model, we obtain $\xi \approx 4\alpha /\left(8+\gamma \right)N^{\delta }$, with δ=−0.98; see Equations (6) and (7). Here, ξ≡ξ(N,α,γ) is the scaling parameter of the model; that is, for fixed ξ, the information entropy of the eigenvectors is also fixed; see Figure 4. Our analysis provides a way to predict the localization properties of the random networks with losses and gain: for ξ<0.1, the eigenvectors are localized; the localization-to-delocalization transition occurs for 0.1<ξ<10; while when 10<ξ, the eigenvectors are extended. Moreover, by recalling that in tight-binding systems, a localization-to-delocalization transition implies an insulator-to-metal transition in the corresponding scattering setup, our results might be used to design the conduction properties of the tight-binding network since tuning *N*, α, and γ could drive the network from a regime of localized eigenvectors (insulating regime), ξ<0.1, to a regime of delocalized eigenvectors (metallic regime), ξ>10.

Therefore, to extend the applicability of our findings, we demonstrate that for fixed ξ, the spectral properties (characterized by the position of the eigenvalues on the complex plane) of our network model are also universal; i.e., they do not depend on the specific values of the network parameters; see Figure 6.

We expect our results may motivate further numerical, as well as analytical efforts towards the understanding of networks with non-Hermitian adjacency matrices. 

## Figures and Tables

**Table 1:** Values of C and δ obtained from the fittings of the curves $\alpha^{*}$ vs. *N* of Figure 3a with Equation (5).

γ	0	0.2	0.4	0.6	0.8	1	1.2	1.4	1.6	1.8	2
C	2.18	2.06	2.09	2.11	2.17	2.27	2.28	2.33	2.4	2.46	2.5
δ	−0.997	−0.982	−0.979	−0.976	−0.976	−0.976	−0.978	−0.977	−0.979	−0.979	−0.979
